# Characteristics of phage-plasmids and their impact on microbial communities

**DOI:** 10.1042/EBC20240014

**Published:** 2024-12-17

**Authors:** Ruweyda Sayid, Anne W.M. van den Hurk, Daniela Rothschild-Rodriguez, Hilde Herrema, Patrick A. de Jonge, Franklin L. Nobrega

**Affiliations:** 1 School of Biological Sciences, University of Southampton, Southampton, UK; 2 Departments of Internal and Experimental Vascular Medicine, Amsterdam UMC, Location AMC, University of Amsterdam, Amsterdam; the Netherlands; 3 Amsterdam Gastroenterology, Endocrinology & Metabolism; Endocrinology, metabolism & nutrition, Amsterdam UMC, Amsterdam, the Netherlands; 4 Amsterdam Cardiovascular Sciences, Diabetes & Metabolism, Amsterdam UMC, Amsterdam, the Netherlands

**Keywords:** Antimicrobial resistance (AMR), Bacteriohages, Horizontal Gene Transfer (HGT), Microbiome, Phage-Plasmids (P-Ps), Plasmids

## Abstract

Bacteria host various foreign genetic elements, most notably plasmids and bacteriophages (or phages). Historically, these two classes were seen as separate, but recent research has shown considerable interplay between them. Phage-plasmids (P-Ps) exhibit characteristics of both phages and plasmids, allowing them to exist extrachromosomally within bacterial hosts as plasmids, but also to infect and lyse bacteria as phages. This dual functionality enables P-Ps to utilize the modes of transmission of both phage and plasmids, facilitating the rapid dissemination of genetic material, including antibiotic resistance and virulence genes, throughout bacterial populations. Additionally, P-Ps have been found to encode toxin-antitoxin and CRISPR-Cas adaptive immune systems, which enhance bacterial survival under stress and provide immunity against other foreign genetic elements. Despite a growing body of literature on P-Ps, large gaps remain in our understanding of their ecological roles and environmental prevalence. This review aims to synthesise existing knowledge and identify research gaps on the impacts of P-Ps on microbial communities.

## Introduction

**Figure 1:**
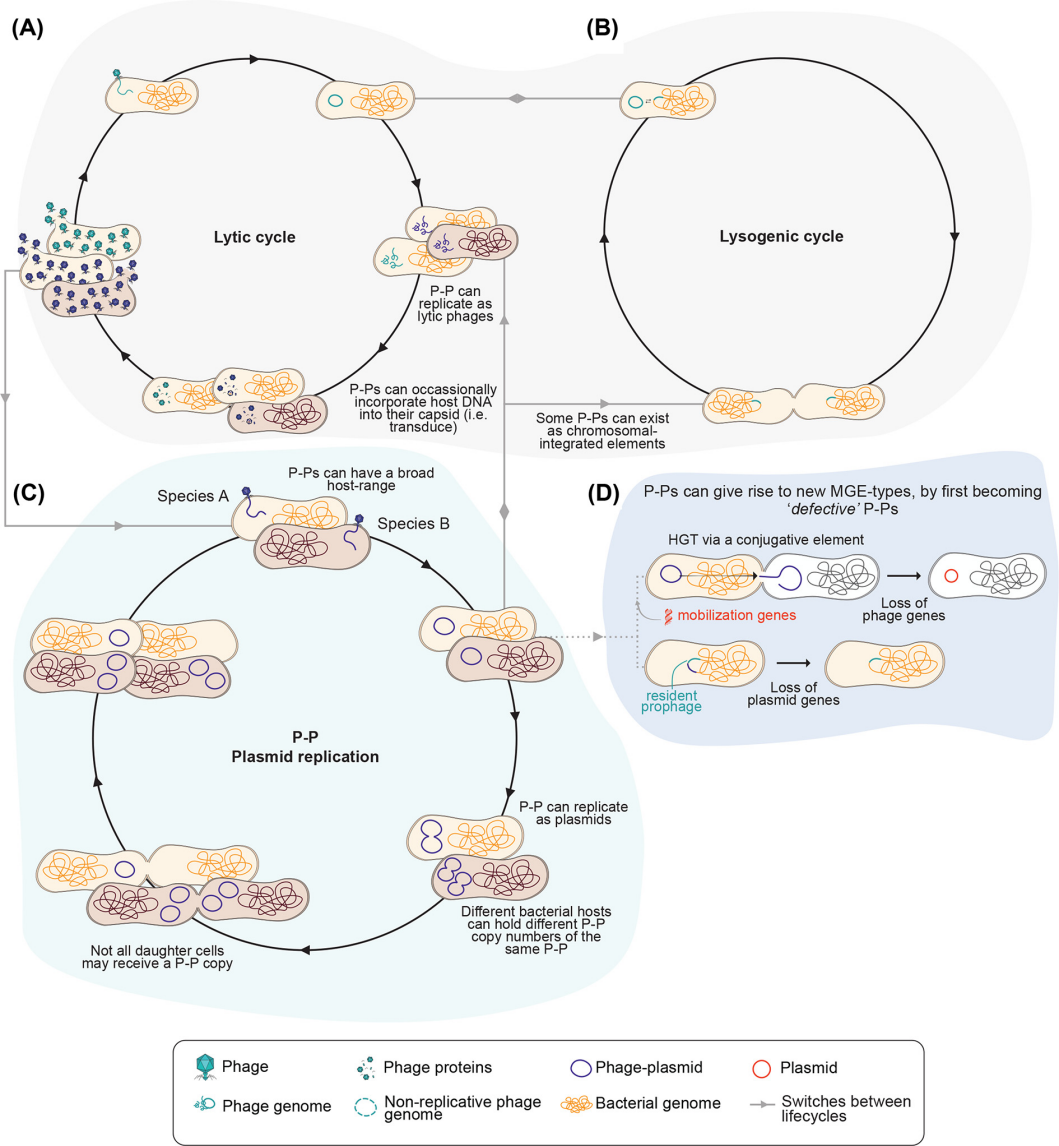
Overview of phage-plasmid (P-P) transitions between phage and plasmid replication strategies (**A**) The lytic lifestyle is characterised by P-P infection that leads directly to genome replication, virion assembly, and host cell lysis to release progeny into the environment. (**B**) The lysogenic lifestyle is characterized by the integration of the P-P genome into the host chromosomal DNA as a prophage, replicating with the host until a trigger causes it to excise and initiate the lytic cycle. (**C**) P-Ps can also replicate as plasmids due to the possession of plasmid homologous genes. (**D**) P-Ps can lose plasmid or phage genes, which can result in the formation of novel elements such as plasmids or phages.

Plasmids and bacteriophages (hereafter phages) are important in shaping bacterial ecosystems [1–6]. Plasmids are extrachromosomal DNA molecules most commonly transferred between bacteria through conjugation; they often encode accessory genes that provide the bacterial host with a selective advantage [1–3]. In contrast, phages are DNA or RNA molecules encapsulated in a proteinaceous capsid that prey on bacteria. Generally, these can be broadly categorised as temperate or virulent phages, which follow lytic and lysogenic life cycles (Figure 1a,b). Temperate phages can integrate their DNA into the bacterial host chromosome potentially adding genes that confer a selective advantage to bacteria [7–9]. The acquisition of a new trait via expression of a phage-derived gene by a bacterial host is known as lysogenic conversion. During the lysogenic life cycle (Figure 1b) of temperate phages, phage DNA is replicated alongside bacterial DNA, and the relationship between phage and bacteria may be described as symbiotic. However, temperate phages can also exhibit parasitic behaviour, particularly if they disrupt essential host genes that interfere with normal cellular functions, or if they do not provide a clear advantage to the host incurring a metabolic burden [10]. Temperate phages also switch to a lytic life cycle – the sole life cycle of virulent phages – where phages hijack host cell machinery to replicate and ultimately lyse the host cell to release viral particles, which then infect neighbouring cells (Figure 1a). As such, both phages and plasmids can drive and shape bacterial ecosystems by affecting the fitness of bacteria, or, in the case of phages, by lysing the host cell. As a result of their differing nature and mechanisms of action, phages and plasmids are considered distinct entities. Phage-plasmids (P-Ps), explored in this review, represent the converged functionality of phages and plasmids.

Phage P1, isolated in the 1950s, is the earliest example of a genetic element exhibiting characteristics of both phages and plasmids and the most studied P-P to date. It was initially identified as a temperate phage [11]. However, later studies discovered its ability to also exist and replicate in a plasmid-like form [12]. Several other elements with similar dual characteristics have been isolated and described since, including N15, SSU5 and D6 [13–15]. While P-Ps were introduced as a distinct mobile genetic element (MGE) class several decades ago [16], they have been overlooked and understudied until recent years, where more of such elements have been discovered and found to play a role in bacterial ecosystems. It is now evident that these MGEs bridge the divide between phages and plasmids, being both similar to, and separate from both these elements [16–18].

The increased understanding on P-Ps lead to the exploration of how P-Ps regulate and transition between the typical phage life cycles (Figure 1). P-Ps carry both phage-homologous genes, such as those that encode viral replication and packaging machinery, and plasmid-homologous genes, such as replication initiators and partition systems. This allows them to exist as genetic elements inside their bacterial hosts (so-called episomes) and replicate as either plasmids or phages (Figure 1c) [18,19]. If replicating as plasmids, variable copy numbers of the P-P can be maintained, depending on the regulating genes they possess and on the P-P’s adaptation to the bacterial host [19,20]. P-Ps maintained as high or low copy number plasmids may influence heterozygosity or homozygosity within bacterial cells of the same lineage [20]. If replicating as a phage, P-Ps can follow the lytic or lysogenic cycle. Sometimes, this leads to transduction of bacterial host DNA [21,22]. Interestingly, P-Ps are also highly mobile elements that undergo high rates of genetic transfer with phages and plasmids [23]. Furthermore, P-Ps can integrate into the host chromosomal DNA, often next to residing prophages, leading to the eventual loss of plasmid genes, becoming a new prophage [23]. Likewise, P-Ps were shown to acquire mobilisation genes (e.g. *mob* and *oriT* genes) that may allow them to mobilise via an autonomously conjugative element and consequently afford to lose phage genes and eventually become a new plasmid (Figure 1d) [19,23]. Nevertheless, P-Ps are suspected to be an ancient type of genetic element and not just a transient state moving between plasmids and phages [18].

Current data, which is mostly centralised around P1 and related P-Ps, show the relevance of these genetic elements for microbial evolution. This review synthesises recent findings from the literature regarding the definition and characteristics of P-Ps, the maintenance of P-Ps within their hosts, their roles in horizontal gene transfer, and their prevalence and influence of microbial communities. We further highlight the need to direct current research efforts towards P-P dynamics and effects, which may differ based on the P-P type, the bacterial host, and the environment it is in.

## Characteristics of phage-plasmids

Increased reporting of MGEs with characteristics of both phages and plasmids initiated a widespread, systematic bioinformatic search for phage gene homologues in plasmids, and plasmid gene homologues in phages to identify putative P-Ps [18]. This search revealed that P-Ps are abundant and widespread, with 7.3% of plasmids and 5.3% of phages in RefSeq being identified as P-Ps. This likely is an underestimation due to conservative searches and RefSeq being biased towards virulent phages. Nevertheless, these estimates represented a total of 780 P-Ps that spanned various bacterial genera [18]. In some bacterial genera, such as *Bacillus* or *Clostridia*, up to half of the phages or plasmids were classified as P-Ps. We expect that P-P identification is bound to increase with the growth of genomic data and increased understanding of them.

**Figure 2:**
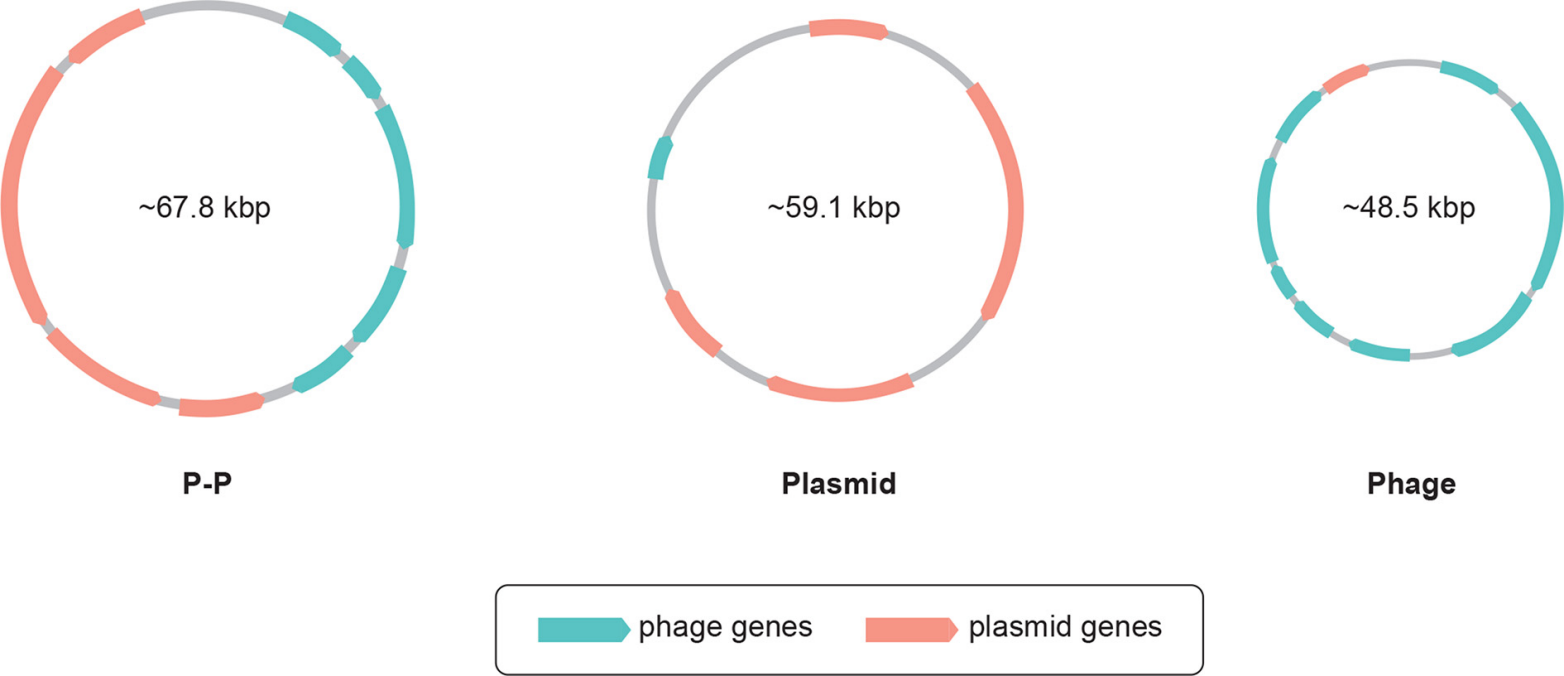
Genomic characteristics of phage-plasmids compared with phages and plasmids Phage-plasmids (P-Ps) are characterised by the presence of both plasmid and phage genes in their genomes and their ability to use both sets of genes for self-replication and better survival. Consequently, P-Ps have a larger genome on average, which results in higher genome plasticity. The number of phage and plasmid genes is indicative. kbp - kilo base pair

Analysis of the identified P-Ps revealed that their genome, which can be circular or linear, is on average 67.8 kbp long with a binomial distribution peaking at around 50 kbp and at around 100 kbp [18]. This is notably larger than the average genome size of plasmids (59.1 kbp) or phages (48.5 kbp) [18]. Due to their averaged larger genomes, P-Ps tend to have higher plasticity than phages [18]. P-Ps generally encode more phage-like genes than plasmid-like genes due to the greater number of essential genes required for phages compared with plasmids (Figure 2). Phage-like functions include lysogeny, DNA replication and packaging, viral morphogenesis, and lysis [24]. Plasmid-like functions include replication and partitioning of the plasmid, plasmid addiction, multimer resolution, and conjugation [25–27]. As noted, P-Ps can lose their phage-homologous or plasmid-homologous genes, evolving into either plasmids or integrated prophages, but the driving factors for this MGE divergence are currently unknown [23]. However, formation of P-Ps from phages or plasmids through acquisition of phage- or plasmid-homologous genes has not yet been observed.

P-Ps have been classified into eight well-defined groups (Table 1), and several other more diverse categories [18]. Among the well-defined groups are the P1, N15, SSU5, and D6 P-Ps [11,13–15]. This classification provides deeper insights into the characteristics and diversity of P-Ps. Firstly, while P-Ps generally contain more phage than plasmid homologous genes, some groups like P1 and pMT1 do not abide this dogma, with each having more plasmid homologous genes instead (phage-plasmid coefficient, Table 1). Notably, an overrepresentation of either phage or plasmid genes does not appear to be representative of P-Ps preferred lifestyle. For example, metatranscriptomic analysis of the canonical crAssphage *Carjivirus communis*, which is suggested to be a P-P, showed that its plasmid genes were more highly expressed than the phage genes, suggesting a preferred plasmid replication strategy, even though it has more phage genes [19]. Secondly, while some P-Ps infect a specific bacterial genus, others can infect several closely related genera, not differing from classical phages [18]. Finally, some P-Ps carry accessory genes such as antibiotic resistance genes [28], which they readily transfer [23]. Overall, these groupings demonstrate that P-Ps are diverse but can be classified based on shared characteristics where enough data is available. Following these classifications can help unify growing data on P-Ps.

**Table 1:** Well-defined P-P groups and their characteristics by Pfeifer and Rocha [18]

Group	Members	Average size	Phage-plasmid coefficient	Inc type	Phage morphology	Bacterial host	Recently transferred genes	AMR genes [28]	Well-known group members
AB	24	111 kbp	0.58	Unknown	*Siphophage*	*Acinetobacter*	9.1%	Yes	
N15	44	55 kbp	0.69	Unknown	*Siphophage*	*Klebsiella, Escherichia, Citrobacter*	18.2%	Not identified	[13,29]
P1	70	95 kbp	0.26	p0111, IncY, IncFII	Myophage	*Escherichia, Salmonella, Shigella*	29.0%	Yes	[12,15,30,31]
pCAV	9	111 kbp	0.69	Unknown	*Siphophage*	*Klebsiella*	16.8%	Not identified	
pKpn	42	111 kbp	0.60	IncFIB, IncA	*Siphophage*	*Klebsiella, Cronobacter*	18.2%	Yes	
pMT1	37	101 kbp	0.38	IncFIB, IncFII	*Siphophage*	*Yersinia*	34.0%	Not identified	
pSLy3	32	110 kbp	0.63	IncFIB	*Siphophage*	*Escherichia, Shigella, Photorhabdus*	25.6%	Yes	
SSU5_pHCM2	41	107 kbp	0.68	IncFIB	*Siphophage*	*Salmonella, Enterobacter, Citrobacter, Klebsiella*	26.6%	Yes	[14]

## Gene transfer by phage-plasmids

**Figure 3:**
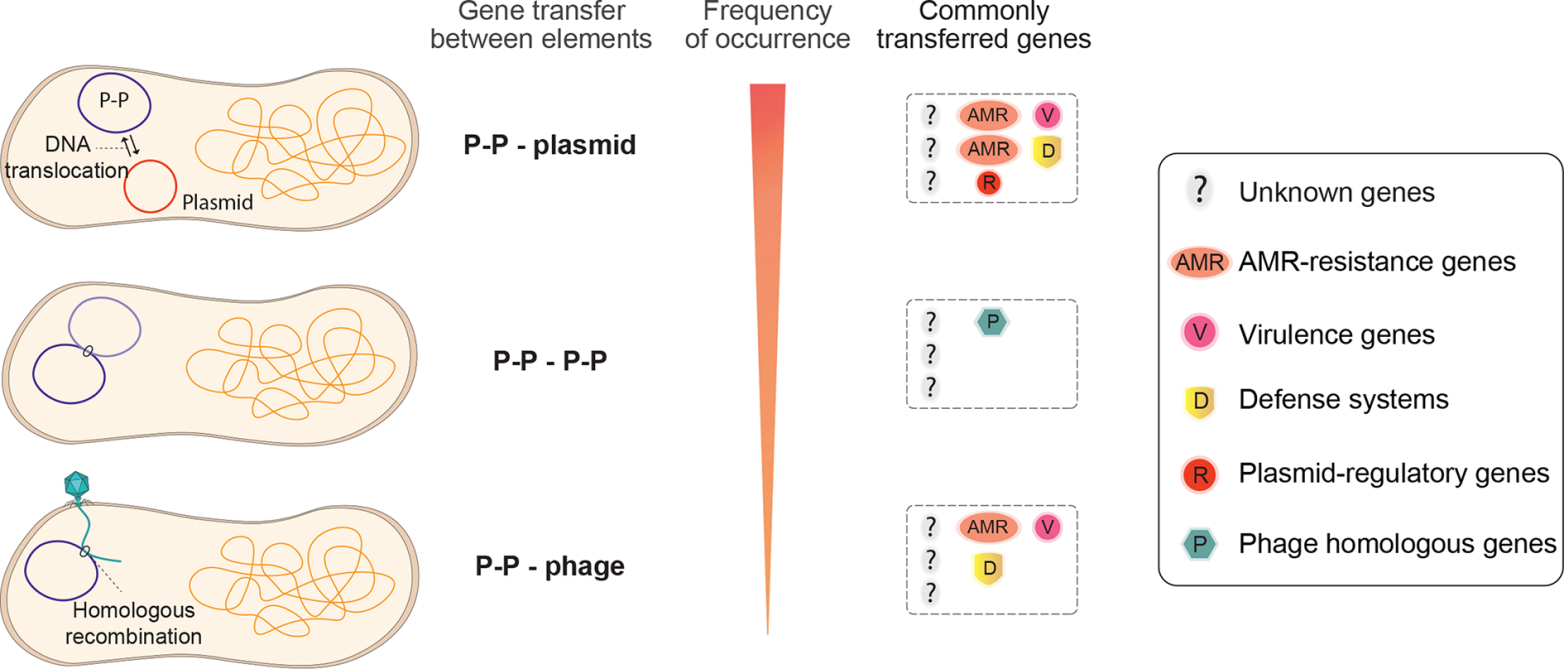
Overview of phage-plasmid-mediated gene transfers Phage-plasmids (P-Ps) undertake gene transfers at a frequency half that of plasmids, but triple that of phages. Within these transfers, they most often exchange genes with plasmids. Most of the genes exchanged by P-Ps are currently of unknown function, but include antimicrobial resistance genes, plasmid-regulatory genes, virulence factors, defence systems, and phage homologous genes (including head, tail and packaging genes). This figure illustrates the findings of Pfeifer and colleagues [23].

The characteristics of P-Ps (i.e. larger genomes and converged homology to phages and plasmids) primes them to facilitate gene transfer between themselves, phages, and plasmids. A study looking at the percentage of recombining genes between these three classes of elements identified that 14.9% of the recombining genes were from P-Ps, compared with 27.1% of genes from plasmids and 4.7% from phages [23]. Interestingly, while direct gene transfer between phages and plasmids is rare, P-Ps more readily exchange genes with both, despite being less abundant (Figure 3). However, the directionality of these gene transfers remains uncertain.

Genes transferred by P-Ps include phage- and plasmid-homologous genes. The same study found that phage homologous genes carried by P-Ps involved in head and packaging were frequently transferred between P-Ps and other phages, while plasmid homologous genes carried by P-Ps were frequently transferred between P-Ps and plasmids (Figure 3). In addition, accessory genes, such as antibiotic resistance genes, virulence factors, and bacterial defence genes like restriction-modification systems were also frequently transferred between P-Ps and plasmids and phages (Figure 3). Interestingly, the majority of genes transferred by P-Ps remain of unknown function, highlighting the complexity of the genetic cargo exchanged between P-Ps and other MGEs.

Gene transfer between P-Ps and plasmids is likely facilitated by transposable elements and recombinases, which are often found near antibiotic resistance genes [28]. Similarly, integrons are common in P1-like and SSU5-like P-Ps [23]. All genes within these integrons in P-Ps were predicted to encode antibiotic resistance [28]. In contrast, recombinases likely play a greater role in gene transfer between P-Ps and phages and among P-Ps, than between P-Ps and plasmids due to the higher sequence homology between the former elements [23]. The transduction capabilities of phages and P-Ps can also facilitate gene transfer via these elements. The virulent phage phi-BB-1 from *Borrelia burgdorferi* can form virions and transduce full-length DNA from the cp32 putative P-P [32] and disseminate its genes across host populations, possibly influencing disease dynamics caused by *B. burgdorferi* [33]. In this instance, both phage phi-BB-1 and P-P cp32 seem to constantly interact within their host to facilitate gene transfer.

Together, these findings suggest that P-Ps frequently mediate gene transfer, especially with plasmids, and to a lesser extent with phages (Figure 3). The nature of P-P lifecycles allows them to rapidly (via phage lytic life cycle), or more passively (as plasmids) disseminate their (newly acquired) genes, such as antibiotic resistance or virulence factors, throughout bacterial populations.

## Persistence and regulation of P-Ps within bacterial hosts

As P-Ps can be integrated into the bacterial genome or exist as independent plasmids, their persistence and regulation involves mechanisms that ensure stable inheritance, countering of MGE infection, and regulation between P-P lifecycles [20,34–38].

Stable inheritance and persistence of P-P are often regulated by partition and toxin-antitoxin (TA) systems. Partition systems ensure that P-Ps are correctly segregated into daughter cells during binary fission, preventing their loss. Typically, these systems are encoded in operons involving genes for an ATPase, a DNA binding protein, and a centromere-like site where the DNA-binding protein attaches [39,40]. Partition systems have been well-studied in P1, where the ParB protein (DNA binding protein) not only binds the *parS* sequence (the centromere-like site) but also ‘spreads’ along adjacent DNA, covering a larger region of DNA beyond the initial binding site [41]. In addition, ParB proteins can oligomerize, forming larger clusters and protein-DNA complexes that are more effectively anchored by cell machinery during cell division [41]. Interestingly, some class A Actinophages possess both integration and ParABS modules. In approximately 20% of cases, the integration module is replaced by the ParABS module, suggesting a potential explanation for how P-Ps can emerge from phages [18,38,42].

P-Ps have also been found to contain TA systems. These systems enhance P-P persistence in the plasmid form by selectively killing cells that lose the plasmid during cell division [40]. Such systems, also known as plasmid addiction modules, usually encode a stable toxin protein and a short-lived antitoxin in the host cell. If the P-P is not passed onto daughter cells during cell division, antitoxin is quickly degraded, allowing the stable toxin to kill the bacteria. In fact, studies have shown that encoding such systems in P1 limits the loss rate of P-P to only 1 in 10^5^ bacterial populations [40].

Protection of P-Ps against other infecting MGEs to ensure their maintenance is achieved by encoding defence systems. These systems were also shown to be commonly exchanged between P-Ps and plasmids, particularly restriction modification systems [23]. Other systems found in P-Ps include PrrC, CBASS, Rst, Mokosh type I and CRISPR-Cas [23,40]. The presence of these systems may also enhance the stability of bacterial populations, particularly under stress conditions, by allowing a subset of the population to survive and others to perish [43]. It is therefore also likely that P-Ps carry anti-defence genes to counteract some of the defence systems that may act against them.

Several P1-like P-Ps were found to contain a *tciABC* operon [44]. The *tciA* gene shares similarity to the *terB* gene that is implicated in tellurite resistance and phage inhibition [44]. The presence of this operon suggests that it provides the P-P with a protective advantage shielding their host from toxic compounds like tellurium salts, which are commonly found in industrial and mining areas where tellurium may be present due to byproducts of copper and gold extractions, and from phage attacks [45].

Other regulatory systems have been identified that control P-P lifecycle switches. Vibrio phage and P-P VP882 uses a quorum sensing receptor that recognises a host-produced autoinducer to switch to the lytic cycle, which is also regulated by sensing of DNA damage (via the cI repressor) [37]. Other quorum sensing mechanisms, like Arbitrium systems, also seem to be associated with putative P-Ps that can regulate switches between lifecycles, specifically through the pheromone receptor AimR [36]. Other regulators include LexA repressors. For instance, P-P GIL01 was shown to at least partially use host LexA-binding as a repressor [46], while N15 has a LexA-regulated anti-repressor for induction [47]. Common DNA-damaging inducers such as UV irradiation and mitomycin C have been shown to induce some P-Ps [28,48].

In summary, P-Ps have evolved various strategies to ensure both their maintenance and that of their host under stressful conditions. Uncovering the factors that shape the regulatory mechanisms employed by different P-P groups, particularly the influence of their host and the surrounding environment, will provide key insights into their evolutionary dynamics.

## Biodiversity of phage-plasmids

Currently, little is known about the prevalence and ecological role of P-Ps across different environments and ecosystems. The widespread presence of plasmids and phages in microbial communities suggests that P-Ps are also likely widespread [15,18,19,23]. Supporting this, P-Ps have been identified in several environments, including the ocean, glaciers, and the mammalian gut [19,20,40].

For example, P-P A3R06 was discovered in a *Tritonibacter mobilis* strain isolated from a marine environment in Massachusetts, USA [20]. Gene homologue searches in other *T. mobilis* genomes found nearly identical plasmid-related gene arrays in a *T. mobilis* P-P from a marine aquaculture environment in Denmark. However, phage-related genes differed. A3R06 P-P phage genes shared high homology with a P-P from a *Roseobacter s*train isolated from the Arabian Sea. This observation was repeated in related P-Ps, indicating that while P-P genome synteny can be preserved in similar environments, the evolution of phage and plasmid genes may be decoupled. At present, it is unknown whether this pattern is unique to A3R06-related P-Ps and which environmental or host factors influence these evolutionary pressures.

In another instance, putative P-Ps were assembled from sequenced samples of Norwegian glaciers [40]. These P-Ps were predicted to have various bacterial species as hosts, including *Actinobacteriota* (*Actinobacteria*)*, Alphaproteobacteria*, and *Bacillota* (*Firmicutes*).

Following the identification of the prototypic crAssphage, *Carjivirus communis*, as a putative P-P [18,19], researchers searched for additional P-Ps within the human-gut associated *Crassvirales* order by searching for *repL* gene homologs in other crAssphages. Despite this effort, only 19 additional putative P-Ps were identified using this approach [19], suggesting that the *repL* gene may be more widespread than currently detected due to high divergence within this order. A broader search that includes both phage- and plasmid-related genes may be more effective in uncovering these elements [18]. Nevertheless, this is the first instance where a widespread gut-associated phage order is known to encompass temperate and virulent phages as well as P-Ps – a finding that will likely be repeated in subsequent years as we learn more about P-Ps. This highlights the potential need to revise phage classification and identification pipelines to differentiate between these elements. This also raises questions about the distinct roles of lytic and temperate phages, as well as P-Ps within the mammalian gut ecosystem [19,49–53].

Moreover, the dominance of P-Ps in gene mobilisation and transfer has led to studies focusing on their association with antimicrobial resistance genes [28,30,31,35,54,55]. This has uncovered several P-Ps within clinically relevant pathogenic strains including *Acinetobacter baumannii*, *Escherichia coli, Klebsiella* spp., *Salmonella enterica*, *Shigella* spp., *Yersinia pestis*, *Borrelia* spp., *Clostridium* spp., *Mycobacterium* spp., amongst others [18,23]. These findings suggest that P-Ps are prevalent in environments with pathogenic strains, including *E. coli* present in chicken meat [56]. It is still unknown however, whether they are more prevalent in pathogenic or non-pathogenic strains.

Given the importance of P-Ps in gene transfer and their potential impact on various ecosystems and pathogenicity, we advocate for increased research focusing on the biodiversity of P-Ps across different ecosystems. Understanding how P-Ps vary in prevalence, function and dynamics in different ecosystems is crucial, as exemplified by the varying roles of prophages in the mammalian gut compared with other environments [49].

## Current challenges in phage-plasmids studies

P-P research faces several challenges, including inconsistent naming, limitations in detection methods, and the absence of comprehensive, searchable databases. These challenges hinder the identification, characterisation, and understanding of P-Ps.

One key issue is the difficulty in detecting P-Ps in genomic data. Current sequencing technologies are biased towards chromosomal DNA [57–60] while P-Ps often exist extrachromosomally. There is also a lack of specialised identification tools for P-Ps. As P1 remains the most well-characterised P-P, other P-Ps have been identified by searching for homologs of P1 genes (*e.g*., major capsid protein [15,61], *repA* [30,31], *repL* [19,31,62]. Broader searches for plasmid genes within phage genomes and *vice versa* have also been reported for P-P identification [18,23], but these efforts are limited by incomplete phage and plasmid gene annotations and fail to account for whether P-Ps are active or dormant (i.e. functional or defective P-Ps).

Additionally, P-Ps may not be culturable via standard plaquing methods [19], further complicating their detection and subsequent implications. Consequently, the true prevalence of P-Ps in various environments is likely underestimated, and their impact remains poorly understood.

Another complication is the inconsistent terminology used in the literature, with P-Ps being referred to by various names such as “episomal phage”, “plasmidial phage”, “extra-chromosomal prophages/plasmids/elements”, “non-integrative prophages/lysogens”, “prophage-plasmids”, amongst other variations. This lack of standardisation makes it difficult to unify findings across studies. To address this, it is crucial to standardise the terminology and adopt a universal term; we propose using “phage-plasmids” (P-Ps). In addition, P-Ps need to be appropriately classified in existing databases [61]. Furthermore, establishing curated databases specifically for P-P genomes would greatly improve research in this area.

## Conclusion

This review highlights the distinct nature of P-Ps as dynamic MGEs that have characteristics of both phages and plasmids. P-Ps as a distinct class of MGE illustrate the complexity of phages that go beyond classical virulent and temperate lifestyles. P-Ps play a crucial role in horizontal gene transfer, facilitating the exchange of accessory genes among bacterial species and other MGEs [19,28,30]. Phylogenetic analyses and computational tools offer promising approaches to better understand the pathways and mechanisms behind gene flow in P-Ps. Further investigation into these processes can help map how P-Ps acquire or lose genes, improving our understanding of microbial evolution.

P-Ps are now known to be widespread and play a role in gene transfers with other MGEs, thus providing sufficient evidence that P-Ps likely shape microbial ecology and evolution. Future research should hence focus on uncovering what these effects might be (e.g. in health vs disease, in pathogenic strains vs commensal vs pathobionts) and their mechanisms (e.g. driving factors for the plasmid-phage lifestyle switches). Developing new methodologies to culture or study P-Ps in laboratory settings will also be key to uncovering their prevalence and functional diversity.

Despite growing interest in P-Ps, their classification remains ambiguous, with many studies still categorising them as either phages or plasmids, which limits their understanding. Recognising P-Ps as a distinct class of MGEs and unifying their terminology is essential to fully study and appreciate their ecological roles and evolutionary flexibility, as demonstrated throughout this review.

## Summary

- Phage-plasmids (P-Ps) are unique mobile genetic elements that combine phage and plasmid characteristics, deserving distinct recognition yet classed as a subtype of both phages and plasmids.
- P-Ps drive microbial evolution and ecology by facilitating horizontal gene transfer between different mobile genetic elements and bacterial species.
- P-Ps can give rise to new genetic elements like plasmids and phages.
- P-P prevalence is likely widespread yet underestimated, with evidence of their existence in diverse environments, from marine ecosystems to the mammalian gut.

## Abbreviations

MGE: mobile genetic element
TA: toxin-antitoxin
